# QTL cluster analysis and marker development for kernel traits based on DArT markers in spring bread wheat (*Triticum aestivum* L.)

**DOI:** 10.3389/fpls.2023.1072233

**Published:** 2023-02-10

**Authors:** Zhankui Zeng, Dehui Zhao, Chunping Wang, Xuefang Yan, Junqiao Song, Peng Chen, Caixia Lan, Ravi P. Singh

**Affiliations:** ^1^ College of Agronomy, Henan University of Science and Technology, Luoyang, Henan, China; ^2^ The Shennong Laboratory, Zhengzhou, Henan, China; ^3^ College of Plant Science and Technology, Huazhong Agricultural University, Wuhan, Hubei, China; ^4^ Global Wheat Program, International Maize and Wheat Improvement Center (CIMMYT), Mexico, Mexico

**Keywords:** QTL mapping, kernel-related traits, putative candidate gene, KASP markers, *Triticum aestivum* L.

## Abstract

Genetic dissection of yield component traits including kernel characteristics is essential for the continuous improvement in wheat yield. In the present study, one recombinant inbred line (RIL) F_6_ population derived from a cross between Avocet and Chilero was used to evaluate the phenotypes of kernel traits of thousand-kernel weight (TKW), kernel length (KL), and kernel width (KW) in four environments at three experimental stations during the 2018–2020 wheat growing seasons. The high-density genetic linkage map was constructed with the diversity arrays technology (DArT) markers and the inclusive composite interval mapping (ICIM) method to identify the quantitative trait loci (QTLs) for TKW, KL, and KW. A total of 48 QTLs for three traits were identified in the RIL population on the 21 chromosomes besides 2A, 4D, and 5B, accounting for 3.00%–33.85% of the phenotypic variances. Based on the physical positions of each QTL, nine stable QTL clusters were identified in the RILs, and among these QTL clusters, *TaTKW-1A* was tightly linked to the DArT marker interval *3950546*–*1213099*, explaining 10.31%–33.85% of the phenotypic variances. A total of 347 high-confidence genes were identified in a 34.74-Mb physical interval. *TraesCS1A02G045300* and *TraesCS1A02G058400* were among the putative candidate genes associated with kernel traits, and they were expressed during grain development. Moreover, we also developed high-throughput kompetitive allele-specific PCR (KASP) markers of *TaTKW-1A*, validated in a natural population of 114 wheat varieties. The study provides a basis for cloning the functional genes underlying the QTL for kernel traits and a practical and accurate marker for molecular breeding.

## 1 Introduction

Wheat (*Triticum aestivum* L.) is one of the most important cereal crops and is a major contributor to the diet of 4.5 billion people worldwide, providing approximately 20% of the daily protein and calorie requirements. Consequently, high yield has long been the primary aim in wheat breeding (Guo et al., 2016; Tao et al., 2018; Michel et al., 2019). Kernel traits are important indicators of wheat yield (Li et al., 2018; Ma et al., 2019); understanding the genes that control these traits can provide a theoretical basis and useful information for wheat breeding (Fatiukha et al., 2020; Xin et al., 2020).

With the development of high-throughput molecular biotechnology and functional genomics, research in yield-related traits is becoming more and more convenient (Saini et al., 2022). DNA sequencing technology and single-nucleotide polymorphism (SNP) markers have been widely used in constructing genetic linkage maps (Qu et al., 2022). In recent years, the successful development of wheat diversity arrays technology (DArT) has dramatically accelerated the research on wheat genetic diversity, gene mapping, and cloning (Ahmed et al., 2021). Grain yield, thousand-kernel weight (TKW), kernel length (KL), and kernel width (KW) are widely known complex quantitative traits, which are controlled by a large number of quantitative trait loci (QTLs)/genes (Simmonds et al., 2016; Wang et al., 2018; Guan et al., 2019; Xu et al., 2019) and environmental influences (Kumar et al., 2016; Kumari et al., 2018). Among such traits, TKW has a high and relatively stable heritability (Kuchel et al., 2007; Sharma et al., 2018); meanwhile, relevant research shows that TKW is influenced by KL and KW (Dholakia et al., 2008; Su et al., 2016). Currently, many genes contributing to grain yield have been identified and cloned in crops, such as *TGW2* (Ruan et al., 2020), *GS3* (Mao et al., 2010), *GW7* (Wang et al., 2015), *TaTPP-6AL1* (Zhang et al., 2017), *TaTGW6* (Hanif et al., 2015), *TaGS-D1* (Zhang et al., 2014), and *TaGS1a* (Guo et al., 2013). A high-yielding gene (*OsDREB1C*), which was detected in rice, is important to improve photosynthetic efficiency and nitrogen use efficiency, increasing more than 30% of the crop yield (Wei et al., 2022). A kernel length gene (*VRT-A2*), which was identified on chromosome 7AS between markers *XP85* and *XP87* with a physical interval of 128.79–128.92 Mb, is a positive regulator of brassinosteroid responses; it encodes an MIKC-type MADS-box protein and significantly increases the kernel length of wheat (Chai et al., 2022).

During the continuous discovery of novel genes, a significant amount of work has been done on gene mining, including QTL mapping, QTL clusters, and pleiotropic QTLs. Many QTLs of kernel traits have been identified on all chromosomes, explaining 0.38%–46.2% of the phenotypic variances (Okamoto et al., 2013; Tyagi et al., 2014; Cheng et al., 2017; Hu et al., 2020; Saini et al., 2022). In addition, some pleiotropic QTLs controlling kernel shape and TKW were discovered on chromosomes 2A, 2B, 2D, 4B, 5B, 5D, and 6A, contributing 3.3%–26.4% of the phenotypic variances (Dholakia et al., 2008; Sun et al., 2008; Ramya et al., 2010; Schierenbeck et al., 2021). Three QTL clusters associated with kernel size were located on chromosomes 1B, 2D, and 6D, accounting for 3.92%–27.78% of the phenotypic variances; the physical position of the QTL clusters is 566.6–583.6 Mb, 481.5–512.8 Mb, and 45.9–73.3 Mb, respectively (Qu et al., 2021). Those gene functions that were associated with kernel traits or kernel weight were mainly affected by three pathways; these pathways are involved in the regulation of cell division and expansion, including phytohormones, G-protein signaling, ubiquitination-mediated proteasomal degradation, and other unknown pathways (Ma et al., 2016; Zhang et al., 2017; Li et al., 2018).

In recent years, with the release of the wheat and closely related species genome sequence, and numerous transcriptome datasets (Duan et al., 2012; Kumar et al., 2015; Yang et al., 2022), all of these might lead to greater convenience for gene mapping, discovery of candidate genes, gene cloning, and development of markers, especially in the area of marker development, such as simple sequence repeat (SSR) markers, cleaved amplified polymorphic sequence (CAPS) markers, kompetitive allele-specific PCR (KASP) markers, and semi-thermal asymmetric reverse PCR (STARP) markers (Wu et al., 2020). The rapid evolution of molecular technology has provided powerful tools to dissect complex traits (Li et al., 2015); many molecular markers for kernel traits have been developed, especially in KASP markers, for example, *KASP-AX-111112626* (tightly linked to kernel length QTL *QKL.sicau-AM-3B*), *KASP-AX-108974756* (tightly linked to kernel width QTL *QKW.sicau-AM-4*B) (Zhou et al., 2021), and *KASP-AX-109379070* (tightly linked to kernel length QTL *QKL.sicau-2SY-1B*) (Qu et al., 2021). The development of these markers has accelerated the rapid development of wheat molecular breeding.

Currently, with the completion of whole-genome sequencing and a fully annotated reference genome of Chinese Spring, and the rapid growth of transcriptomic technologies, the candidate genes can be more conveniently identified and characterized with the help of multiple technologies. The present study is yet another effort to identify the new QTLs of kernel traits and the following related aspects: (1) finding the QTLs for kernel traits, (2) exploring the stable and novel QTL clusters, (3) identifying candidate genes by multiple sequence alignments and gene annotation, and (4) developing KASP markers of the major loci for breeders in breeding programs. We believe that these results should provide useful information not only for molecular breeding but also for basic research on fine mapping and cloning of QTLs in wheat or in other cereals.

## 2 Materials and methods

### 2.1 Plant materials

The recombinant inbred line (RIL) population of 163 F_6_ lines was used for QTL analysis of kernel traits in this study, derived from the cross Avocet × Chilero using the single seed descent approach (Basnet et al., 2014). Chilero had significantly higher values (*p* < 0.05) for all investigated kernel traits than those of Avocet. The International Maize and Wheat Improvement Center (CIMMYT) developed the RIL population.

A natural population with 114 cultivars was utilized for validation of the KASP markers, including 53 wheat accessions collected within the country and 61 cultivars from other countries, such as those in Europe, USA, Mexico, and Australia. Materials were provided by the wheat germplasm innovation and molecular breeding project of Henan University of Science and Technology, China.

### 2.2 Field trials

For phenotyping, the RIL population and parents were grown under four environments at three experimental stations in three seasons: (1) a test field at the farm of Henan University of Science and Technology (34°C60N″, 112°C42E″), during the autumn–winter cycle of 2018–2019 (2018XN) and 2020–2021 (2020XN) cropping seasons; (2) an open field at Luoning county (34°C42″N, 111°C67″E) in the 2019–2020 (2019LN) cropping season; and (3) a test field at Mengjin county (34°C83″N, 112°C58″E) during the 2020–2021 (2020MJ) cropping season. The natural population of 114 varieties or lines was planted in the three experimental stations at Mengjin county and the farm of Henan University of Science and Technology during 2019–2021.

Each trial of both populations was arranged following a randomized block design with three replicates; the lines of the RIL population and the natural population were grown in a 6.0 m^2^ plot at each location, with 10 rows, 20 cm apart and 3.0 m in length for each plot. Field management followed the local agronomic practices.

### 2.3 Phenotypic evaluation

In each experiment, plants were chosen to be harvested after they were completely matured, in order to avoid other factors that may affect the phenotypic analyses. Meanwhile, seeds were dried before analysis. Data parameters of kernel traits were evaluated when all the kernels had approximately 11% moisture content.

The phenotypic values of two populations were determined using the same method. Three kernel traits (TKW, KL, and KW) were measured by using Wanshen SC-A automatic testing equipment, which was developed by Wanshen Science and Technology Ltd. (Hangzhou, Zhejiang, China; www.wseen.com). At least 300 kernels from each line were measured with three replicates, and the average of the three replicates was taken as the evaluation result of each line.

### 2.4 Phenotypic statistical analysis

For each phenotypic trait, SPSS version 22.0 software (SPSS, Chicago, USA) was used to conduct statistical analysis of phenotypic data, including the means, standard deviation (SD), range, kurtosis, skewness, and coefficient of variation. The QTL IciMapping v4.1 software was used to compute the broad-sense heritability (H^2^) and to calculate the best linear unbiased estimate (BLUE) of each kernel trait. The OriginPro 22b software was used to draw the histograms and correlation plot, and the linkage map was drawn using Mapchart.

### 2.5 Quantitative trait locus mapping and candidate gene analysis

A total of 23,526 DArTSeq markers were genotyped for both parents and the RIL population, and the QTL IciMapping v4.1 software was used to construct the genetic linkage map and identify significant QTLs (http://www.isbreeding.net) (Zeng et al., 2020). The Kosambi mapping function was used to calculate centiMorgan units (cM), and the inclusive composite interval mapping (ICIM) method was performed for QTL analysis. For all significant QTLs, the critical LOD values were set at 3.0 to increase the reliability and accuracy of QTL detection, and the walking speed parameter of each step for the genome-wide scan was set at 1.0 cM; the significance thresholds were calculated using 1,000 permutations, with genome-wide error rates of 0.10 and a type I error of 0.01. The naming of QTL followed the rule “QTL + trait + research department + chromosome”. The QTLs detected in two or more environments are considered as stable QTLs (Ruan et al., 2021).

In this study, we performed the candidate gene analysis for a highly significant and stable region on wheat chromosome 1AS (http://plants.ensembl.org/index.html). The high-confidence genes were extracted from the IWGSC reference genome and identified using IWGSC RefSeq v1.1 (https://urgi.versailles.inra.fr/) annotation for the identification of likely candidates.

### 2.6 KASP assay design and genotyping

Based on the mapping results, the sequences flanking the QTL *TaTKW-1A* were used for designing KASP primers (composed of the two forward primers and the reverse primer) (PolyMarker, http://polymarker.tgac.ac.uk/). The primers were synthesized by Sangon Biotech (Shanghai) Co., Ltd. (China).

All KASP reactions were performed in a 4-μl reaction volume, which included 2 μl of diluted DNA, 2 μl of KASP master mix, and 0.045 μl of primer mix. A total of 114 wheat varieties were genotyped on an CFX 384 Real-Time System (BIO-RAD). The fluorescence signals of each reaction well were collected and genotyping was performed using the BioRad CFX Manager Software.

## 3 Results

### 3.1 Phenotypic variation

In the four field trials conducted, the means, standard deviation (SD), range, kurtosis, skewness, and coefficient of variation for each of the phenotypes were calculated in the RIL population. The parental genotype Chilero of the mapping population consistently had significantly higher mean values (*p* < 0.05) for all investigated kernel traits than those of Avocet (**Table 1**). According to the phenotypic distribution, TKW was larger than KL and KW on the range of variation, and the scores of skewness and kurtosis were mostly less than 1.0 for all kernel traits in the four field trials, indicating that they were quantitative traits controlled by multiple genes. All kernel traits had broad-sense heritability higher than 90% (**Table 1**).

**Table 1:** Descriptive statistics of the parental genotypes and RILs for different kernel traits in four environments.

Trait	Environment	Avocet	Chilero	RILs					H^2^
		Mean ± SD	Mean ± SD	Mean ± SD	Range	Kurtosis	Skewness	CV%	
TKW	2018XN	45.490 ± 0.921a	53.824 ± 0.748b	47.098 ± 3.912	38.216–57.688	−0.207	0.172	8.305	0.91
	2019LN	32.427 ± 0.066a	43.981 ± 0.172b	41.328 ± 3.874	30.836–51.208	−0.384	−0.196	9.374	
	2020XN	36.637 ± 0.285a	43.233 ± 0.779b	42.279 ± 3.665	32.894–50.801	−0.199	−0.015	8.669	
	2020MJ	37.643 ± 0.259a	45.096 ± 1.178b	41.179 ± 4.849	29.131–52.728	−0.460	−0.121	11.774	
	Mean	38.035	46.245	43.611 ± 3.305	35.225–52.053	0.088	0.080	7.578	
	BLUE	34.630	45.340	42.252 ± 3.618	32.552–51.326	−0.248	−0.188	8.562	
KL	2018XN	6.664 ± 0.021a	6.855 ± 0.026b	6.697 ± 0.277	6.014–7.421	−0.308	0.158	4.141	0.95
	2019LN	5.952 ± 0.007a	6.281 ± 0.007b	6.200 ± 0.234	5.587–6.769	−0.448	−0.102	3.779	
	2020XN	5.542 ± 0.031a	5.720 ± 0.022b	5.713 ± 0.249	5.267–6.421	−0.109	0.520	4.353	
	2020MJ	5.487 ± 0.022a	5.837 ± 0.051b	5.647 ± 0.208	5.137–6.153	−0.032	−0.075	3.687	
	Mean	5.915	6.159	6.263 ± 0.242	5.752–7.098	0.202	0.378	3.864	
	BLUE	6.000	6.300	6.251 ± 0.240	5.695–7.070	0.047	0.193	3.843	
KW	2018XN	3.127 ± 0.036a	3.441 ± 0.033b	3.240 ± 0.118	2.940–3.538	−0.062	−0.026	3.636	0.91
	2019LN	2.631 ± 0.002a	3.024 ± 0.005b	2.913 ± 0.135	2.503–3.165	−0.266	−0.431	4.647	
	2020XN	2.638 ± 0.018a	2.846 ± 0.016b	2.801 ± 0.102	2.550–3.030	−0.279	−0.044	3.643	
	2020MJ	2.681 ± 0.012a	2.880 ± 0.038b	2.761 ± 0.147	2.387–3.189	−0.067	−0.006	5.326	
	Mean	2.765	3.040	3.001 ± 0.112	2.701–3.347	0.454	−0.039	3.732	
	BLUE	2.710	3.080	2.967 ± 0.127	2.593–3.326	0.029	−0.322	4.284	

Different letters indicate significant differences between the two parents at *p* = 0.05. H^2^: the broad-sense heritability.

**Figure 1:**
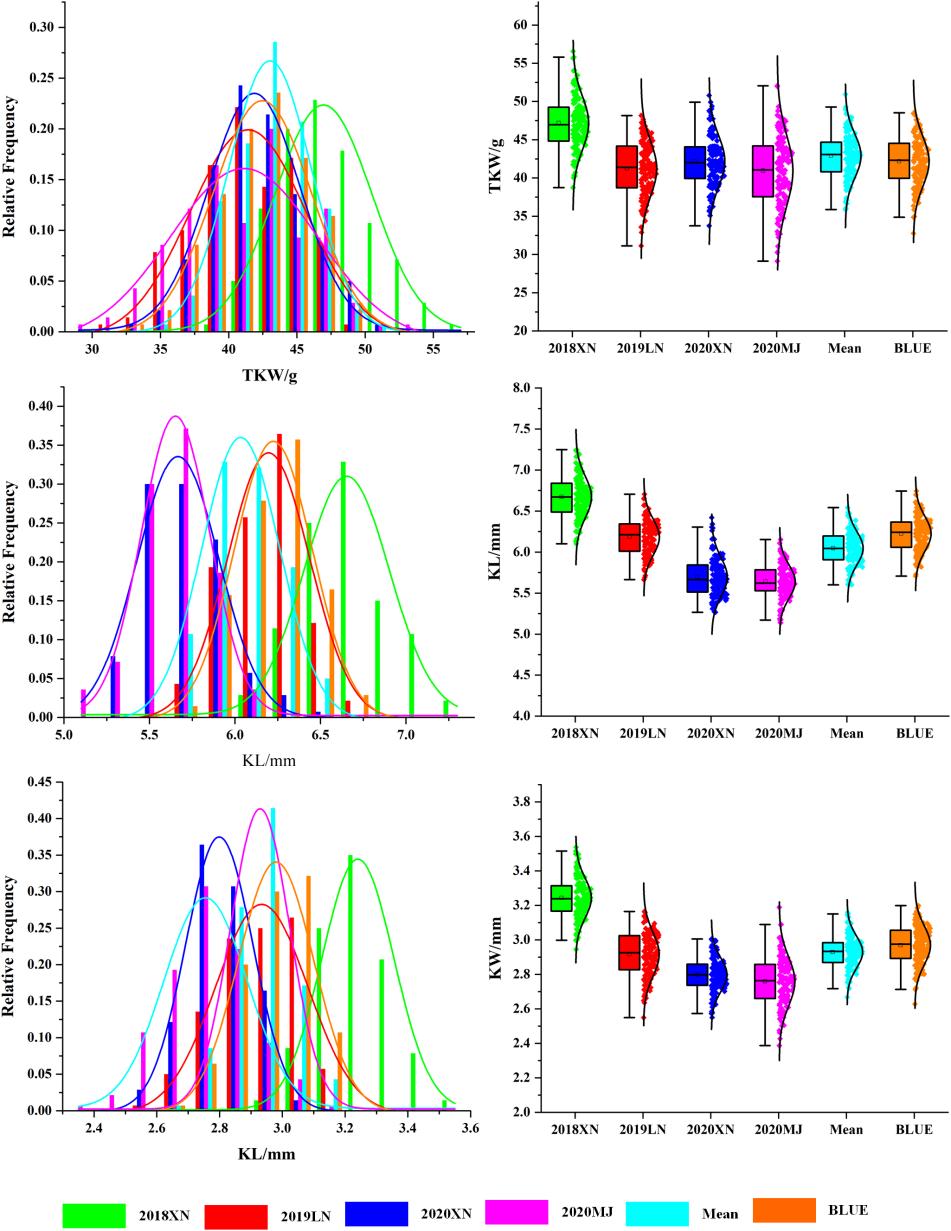
Histograms of distributions for wheat kernel traits in the Avocet/Chilero RIL population. Note: 2018XN: The farm of Henan University of Science and Technology 2018–2019, 2019LN: Luoning 2019–2020, 2020XN: The farm of Henan University of Science and Technology 2020–2021, 2020MJ: Mengjin 2020–2021. Green represents 2018XN, red represents 2019LN, blue represents 2020XN, magenta represents 2020MJ, cyan represents mean values, and orange represents BLUE.

**Figure 2:**
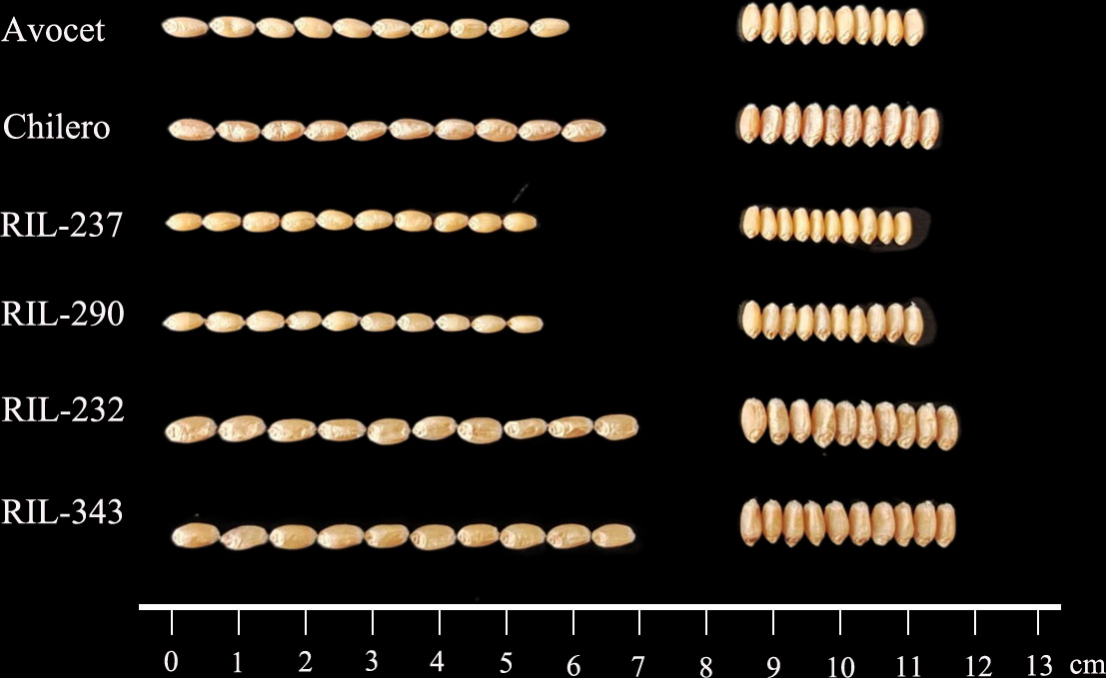
Kernel phenotypes of the parents and lines in the Avocet/Chilero RIL population.

**Figure 3:**
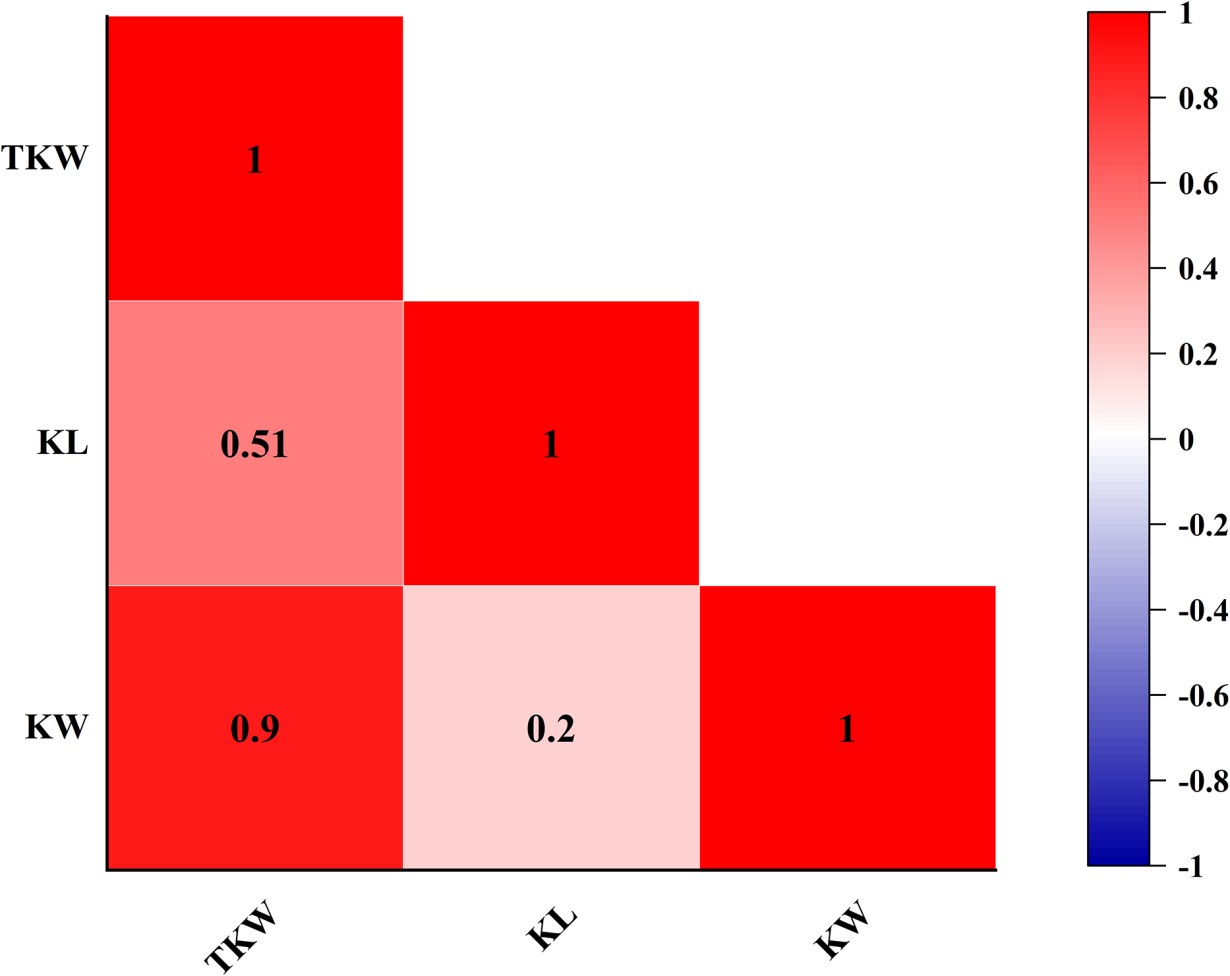
Heat map of Pearson’s correlation of fructan content by using BLUE values.

Continuous variations and strong transgressive segregations have been shown for all three traits in the RIL population, suggesting that favorable alleles of these traits are distributed in both parents, and indicating segregation patterns of quantitative traits (**Figures 1**, **2**). Correlations among kernel traits are significant (**Figure 3**).

### 3.2 QTL identification for kernel traits

In this study, we obtained the results using the ICIM method; a total of 48 QTLs for TKW, KL, and KW were detected and mapped on chromosomes 1A, 1B, 1D, 2B, 2D, 3A, 3B, 3D, 4A, 4B, 5A, 5D, 6A, 6B, 6D, 7A, 7B, and 7D (Tables S1, S2, S3; Figures S1, S2, S3), including 12 QTLs for TKW, 18 QTLs for KL, and 18 QTLs for KW, individually explaining 3.00%–33.85% of the phenotypic variance. Among these QTLs, 9 QTLs for TKW, 12 QTLs for KL, and 13 QTLs for KW explained >10% of the phenotypic variance and were considered the major QTLs.

### 3.3 Identification of the stable QTL clusters

**Figure 4:**
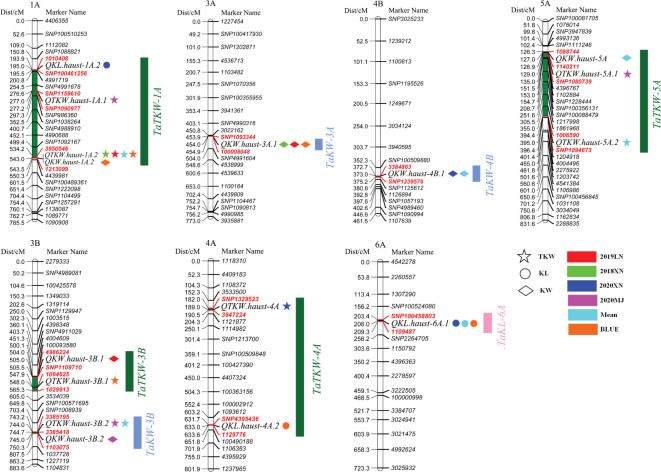
Locations of QTLs of the stable QTL clusters.

The sequences of the flanking markers of the QTLs were employed to perform BLASTN against the Chinese Spring reference genome sequence v1.1. According to the physical location of the QTLs, we identified the stable QTL clusters in the Avocet/Chilero RIL population; the detailed information is described in **Table 2** and **Figure 4**.

**Table 2:** The stable QTL clusters in the Avocet/Chilero RIL population.

No.	Locus	QTL	Environment	Physical position/	Marker interval	LOD	PVE (%)	Add
				Mb				
1	*TaTKW-1A*	*QTKW.haust-1A.1*	2020MJ	14.67–20.87	*SNP1158610–SNP1090977*	5.67	14.61	2.02
		*QTKW.haust-1A.2*	2018XN	14.56–49.30	*3950546–1213099*	21.29	27.57	2.82
			2019LN			9.61	24.02	2.00
			Mean			16.32	33.85	2.12
			BLUE			16.43	31.71	2.29
		*QKW.haust-1A.2*	BLUE	14.56–49.30	*3950546–1213099*	9.15	19.32	0.06
		*QKL.haust-1A.2*	2020XN	39.51–44.36	*1010408–SNP100461256*	4.01	10.31	0.08
2	*TaKW-3A*	*QKW.haust-3A.1*	2019LN	69.50–69.51	*SNP1093344–100008048*	6.84	20.42	0.07
			BLUE			8.24	16.86	0.07
			2018XN			5.92	7.76	0.05
3	*TaTKW-3B*	*QKW.haust-3B.1*	2019LN	17.97–18.00	*4986224–SNP1109710*	3.82	10.60	−0.04
		*QTKW.haust-3B.1*	BLUE	7.29–31.66	*1094625–1029913*	7.39	11.46	−1.53
4	*TaKW-3B*	*QTKW.haust-3B.2*	Mean	614.57–614.57	*3385195–3385418*	3.78	6.02	−0.89
			2020MJ			3.07	7.55	−1.41
		*QKW.haust-3B.2*	2020MJ	490.89–614.57	*3385418–1103075*	5.25	8.18	−0.06
5	*TaTKW-4A*	*QTKW.haust-4A*	2020XN	584.41–606.37	*SNP1329523–3947224*	8.49	20.76	2.24
		*QKL.haust-4A.2*	BLUE	597.08–602.25	*SNP4395436–1129776*	3.5	7.34	0.08
6	*TaKW-4B*	*QKW.haust-4B.1*	Mean	629.70–630.13	*3384863–SNP1239576*	8.69	19.58	−0.05
			2020XN			6.17	20.36	−0.05
7	*TaTKW-5A*	*QKW.haust-5A*	Mean	481.85–481.93	*1088744–1140211*	8.55	19.63	−0.05
		*QTKW.haust-5A.1*	Mean	436.63–481.85	*1140211–SNP1080739*	8.16	13.91	−1.35
		*QTKW.haust-5A.2*	2020MJ	442.50–464.19	*1006590–SNP1094673*	5.03	13.22	−2.18
8	*TaKL-6A*	*QKL.haust-6A.1*	2020XN	61.21–65.66	*SNP100458803–1109487*	6.08	17.52	0.10
			BLUE			6.26	14.15	0.10
			Mean			3.10	7.07	0.07

2018XN: The farm of Henan University of Science and Technology 2018–2019, 2019LN: Luoning 2019–2020, 2020XN: The farm of Henan University of Science and Technology 2020–2021, 2020MJ: Mengjin 2020–2021, Mean: mean values, BLUE: The best linear unbiased estimate. LOD: maximum-likelihood LOD score. Add: ± Additive effect, the positive and negative values indicate contribution of Avocet and Chilero alleles to the larger values, respectively. PVE (%) = phenotypic variance.

The results showed that there were eight stable QTL clusters in the population, and they were renamed *TaTKW-1A*, *TaKW-3A*, *TaTKW-3B*, *TaKW-3B*, *TaTKW-4A*, *TaKW-4B*, *TaTKW-5A*, and *TaKL-6A*. Among the eight stable QTL clusters, four QTL clusters have the favorable allele from Avocet, *TaTKW-1A*, *TaKW-3A*, *TaTKW-4A*, and *TaKL-6A*, and four QTL clusters have the favorable allele from Chilero, *TaTKW-3B*, *TaKW-3B*, *TaKW-4B*, and *TaTKW-5A*. *TaTKW-1A* was closely linked to DArT markers *SNP1158610*, *SNP1090977*, *1010408*, *SNP100461256*, *3950546*, and *1213099* in the physical interval of 14.56–49.30 Mb, explaining 10.31%–33.85% of the phenotypic variance. *TaKW-3A* was tightly linked to marker intervals *SNP1093344*–*100008048* with a physical interval of 69.50–69.51 Mb, accounting for 7.76%–20.42% of the phenotypic variance.

### 3.4 Identification of candidate genes within the *TaTKW-1A* physical interval

**Figure 5:**
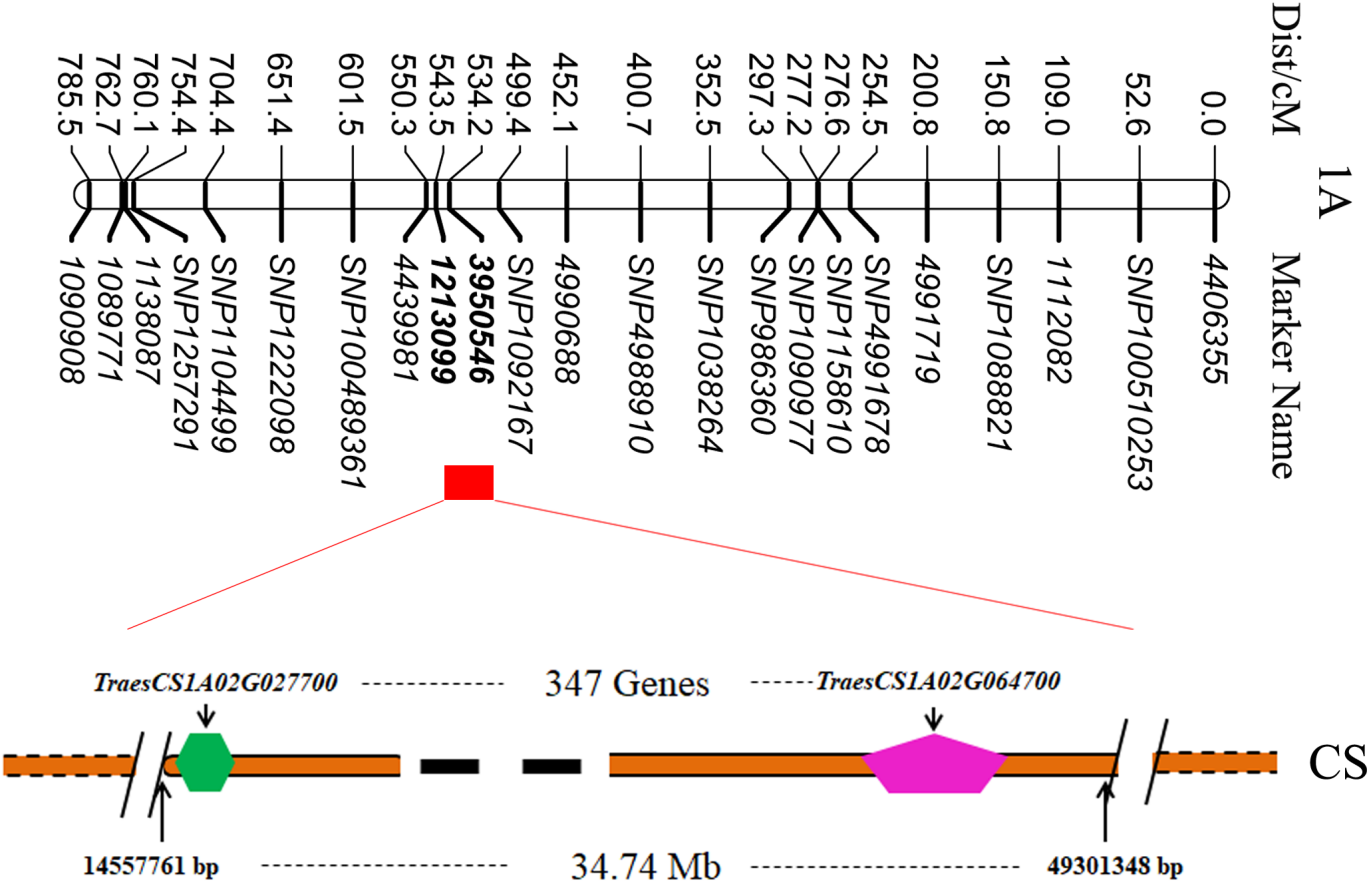
Possible physical segments of candidate genes on 1A chromosome. CS, Chinese Spring.

To clarify the physical position of *TaTKW-1A*, the DArT marker sequence was subjected to alignment with the whole-genome database of Chinese Spring (https://wheat-urgi.versailles.inra.fr/) by using the BLAST tool. Sequence comparison revealed that *TaTKW-1A* was in a physical interval from 14557761 to 49301348 bp on chromosome 1A (**Figure 5**). A total of 347 high-confidence genes with a physical length of 34.74 Mb were identified in the DarT marker interval *3950546*–*1213099* (https://www.wheatgmap.org/).

### 3.5 KASP marker development of *TaTKW-1A*

**Figure 6:**
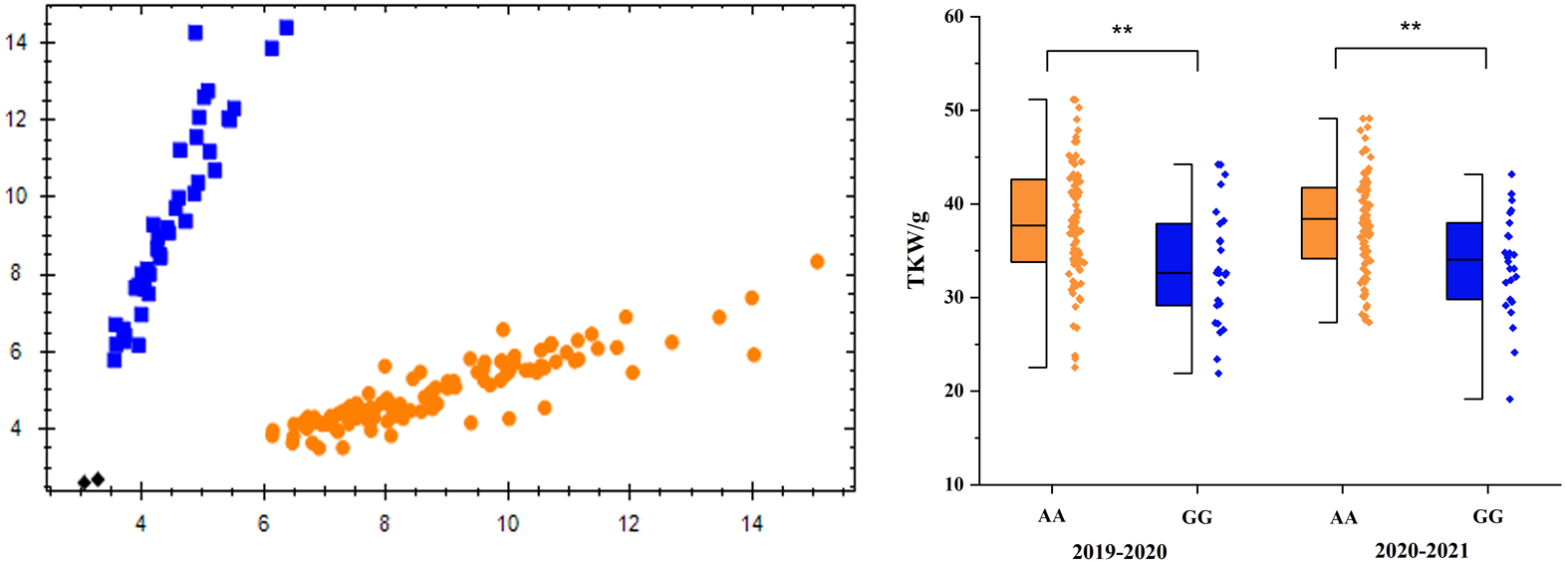
Genotype calling screenshots of the KASP markers. Blue indicates the G allele of Chilero, orange indicates the A allele of Avocet, and black indicates the blank control, **significant at *p* < 0.01. The same below.

For the effective utilization of the major QTL in plant breeding, KASP markers closely linked to TaTKW-1A were developed and used to genotype 114 lines (**Table 3**, **Figure 6**).

**Table 3:** The KASP molecular marker sequence of *TaTKW-1A*.

Locus	Molecular marker	Alleles	Primer sequence (5′-3′)
*TaTKW-1A*	*KASP-1A-A*	A/G	GAAGGTGACCAAGTTCATGCTGCTCGAAACACACTCCGAATAA
	*KASP-1A-B*		GAAGGTCGGAGTCAACGGATTGCTCGAAACACACTCCGAATAG
	*KASP-1A-C*		CCAGTTAGGAGGTCATTGGGC

The underlined part represents the fluorescent junction sequence. A and B in primer names indicate Avocet and Chilero allele-specific primers, respectively, and C indicates common primer.

Of the 114 accessions, there were 26 GG (22.8%) genotypes and 88 AA (77.2%) genotypes (**Figure 6**), and TKW was significantly different (*p* < 0.01) between the two genotypes; in addition, AA (Avocet) genotypes were higher than GG (Chilero) genotypes. Furthermore, 15 wheat varieties have genotype GG and 38 have genotype AA in the 53 domestic wheat accessions, and 11 have genotype GG and 50 have genotype AA in the 61 foreign varieties (Figure S4).

## 4 Discussion

### 4.1 QTLs for kernel traits

Wheat has a very huge and complex genome; QTL mapping can provide important information regarding the molecular basis of determination of kernel-related traits. In past decades, more than 400 QTLs for TKW and approximately 200 QTLs for KL and KW have been reported across all 21 chromosomes, and some stable and robust QTLs were detected (Saini et al., 2022). As a whole, these QTLs were mostly distributed across the A and B genomes as compared to the D genome (Yang et al., 2020). A similar trend was observed in this study, with more QTLs on the A (24) and B (14) genomes than on the D (10) genome. Although many QTLs associated with kernel traits have been identified, its application is rarely reported in molecular marker-assisted breeding (MAS), due to the fact that many QTL locations were based on genetic distances rather than the physical distances.

TKW was a complex quantitative trait that was affected by polygenes. Studies indicated that TKW increased gradually when KL and KW increased, and TKW had a significant positive correlation with KL and KW (Gao et al., 2015; Cui et al., 2016; Chai et al., 2022). In this study, the significant correlation is found between TKW and KL, between TKW and KW, and between KL and KW, with the Pearson correlation coefficients of 0.51, 0.90, and 0.20, respectively, which was consistent with the conclusions of other studies (Liu et al., 2014; Michel et al., 2019; Qu et al., 2022).

### 4.2 QTL cluster analysis for TKW

TKW was a complex polygenic trait with high broad-sense heritability and was less affected by the environment (Cuthbert et al., 2008; Mcintyre et al., 2010; Gao et al., 2015), and QTLs for TKW have been reported on all 21 wheat chromosomes (Huang et al., 2006; Li et al., 2007; Sun et al., 2008; Ramya et al., 2010; Zhang et al., 2014; Shukla et al., 2015; Yu et al., 2018; Chen et al., 2020). In this study, we detected four QTL clusters for TKW, designated *TaTKW-1A*, *TaTKW-3B*, *TaTKW-4A*, and *TaTKW-5A*. Then, based on the sequence information of the markers flanking these QTLs, we found some genes within each of these QTL intervals by using the BLAST tool.

In earlier studies, many QTLs for TKW were reported on 1A (Pinto et al., 2010; Ramya et al., 2010; Williams and Sorrells, 2014; Wu et al., 2015; Cui et al., 2016; Assanga et al., 2017), 3B (Russo et al., 2014; Xu et al., 2014; Shirdelmoghanloo et al., 2016; Li et al., 2018), 4A (Zou et al., 2017; Li et al., 2020), and 5A (Liu et al., 2014; Fowler et al., 2016; Kumar et al., 2016; Assanga et al., 2017; Brinton et al., 2017). Among the four loci, *TaTKW-1A* colocalized with *QTKW.haust-1A.1*, *QKW.haust-1A.2*, *QTKW.haust-1A.2*, and *QKL.haust-1A.2*, with a corresponding physical position of 14557761–49301348 bp on the short arm of chromosome 1A. It overlapped with few published QTLs, such as *QGw.ccsu-1A.1* (physical position: 27.27 Mb), *QTkw.ncl-1A.1* (physical position: 27.27 Mb), and *qTgw.nwipb-1AS* (physical interval: 20.02–25.31 Mb), which was detected by Kumar et al. (2006); Ramya et al. (2010), and Liu et al. (2020a), respectively. However, these QTLs were only detected in one environment, and further studies of the locus have not been reported in the literature. *TaTKW-4A* colocalized with *QTKW.haust-4A* and *QKL.haust-4A.2*. The physical interval (584.41–606.37 Mb) of the loci was not identical to that previously identified (Maphosa et al., 2014; Gao et al., 2015; Chen et al., 2020; Li et al., 2020); these loci may be novel loci for TKW. Another colocated QTL, *TaTKW-5A* (*QTKW.haust-5A.1*, *QTKW.haust-5A.2*, and *QKW.haust-5A*) at 436.63–481.93 Mb, does not coincide with the physical position of other reported QTLs (Wang et al., 2009; Yuan et al., 2017; Guan et al., 2018). *TaTKW-3B* colocalized with *QKW.haust-3B.1* and *QTKW.haust-3B.1*, and it was localized to the physical interval 7.29–31.66 Mb. We have undertaken a detailed analysis of the loci identified on chromosome 3B in previous studies (Huang et al., 2004; Maphosa et al., 2014; Echeverry-Solarte et al., 2015; Shi et al., 2017; Duan et al., 2020); *TaTKW-3B* was different from those QTLs reported on 3B, and was likely a new locus.

### 4.3 QTL cluster analysis for KL and KW

With respect to KL and KW, many QTLs for KL and KW were previously reported on chromosomes 3A, 3B, 4B, and 6A (Breseghello and Sorrells, 2006; Gegas et al., 2010; Cui et al., 2016; Cheng et al., 2017; Li et al., 2018; Zhai et al., 2018). In our study, we detected one QTL cluster for KL on chromosome 6A (*TaKL-6A*, physical interval: 61.21–65.66 Mb) and three QTLs for KW on chromosomes 3A (*TaKW-3A*, physical interval: 69.5–69.51 Mb), 3B (*TaKW-3B*, physical interval: 490.89–614.57 Mb), and 4B (*TaKW-4B*, physical interval: 629.7–630.13 Mb). To confirm whether we identified four loci, a comparative analysis of the physical positions of previously reported QTLs with those identified in this study was conducted. According to the physical intervals of *TaKL-6A*, *TaKW-3A*, *TaKW-3B*, and *TaKW-4B*, we speculated that the four loci were new QTLs (Cui et al., 2011; Cui et al., 2014; Li et al., 2018; Su et al., 2018; Chen et al., 2020; Duan et al., 2020; Xin et al., 2020).

### 4.4 Identification of putative candidate genes for TKW

In recent years, with the rapid development of sequencing technology and bioinformatics, a fully annotated reference genome of Chinese Spring was released (IWGSC RefSeq v1.1, https://urgi.versailles.inra.fr/blast_iwgsc/blast.php), providing a better approach in searching for candidate genes. Meanwhile, due to the co-linearity with grasses and the conservation of gene function among different species, many functional markers in wheat have been developed for many cloned genes of kernel traits.

**Figure 7:**
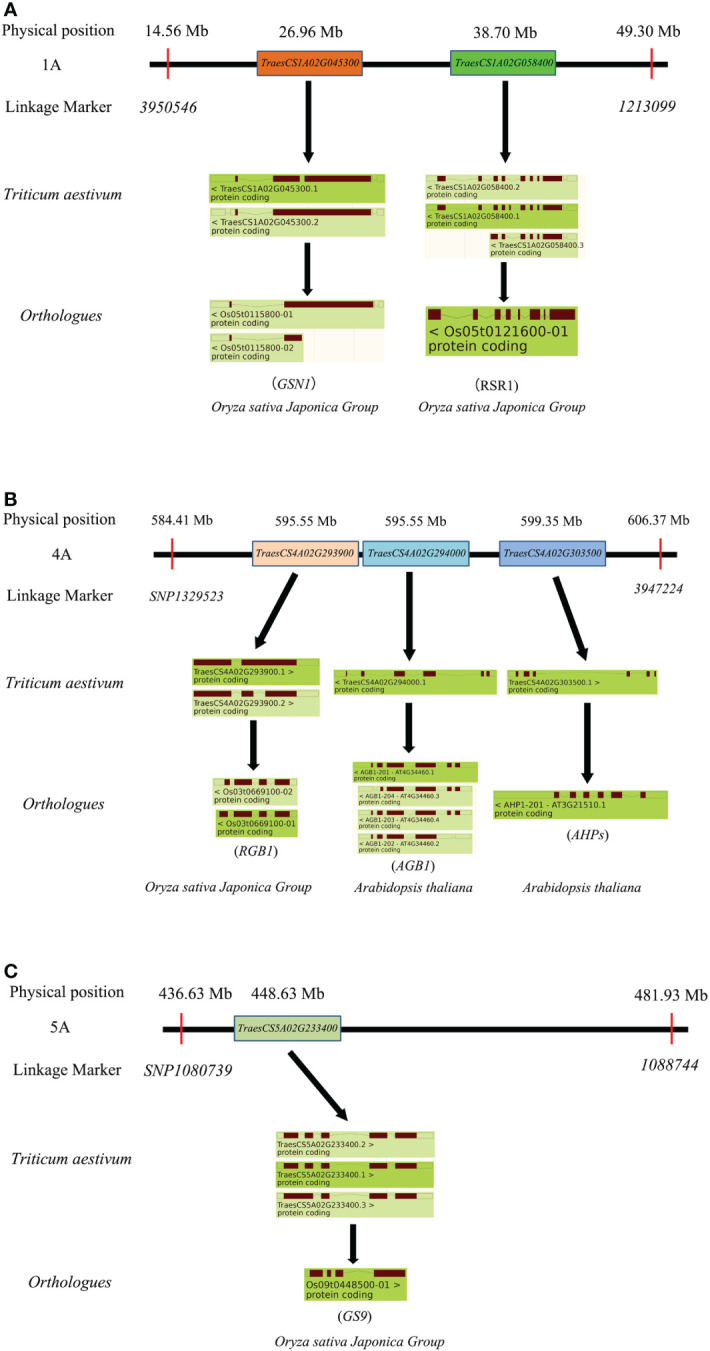
Cloned genes affecting kernel traits in candidate intervals. **(A)** Cloned gene of *TaTKW-1A*. **(B)** Cloned gene of *TaTKW-4A*. **(C)** Cloned gene of *TaTKW-5A*.

In *TaTKW-1A*, blasting results showed a physical interval of 14.56–49.30 Mb, and a total of 347 high-confidence genes were found (**Figure 5**). Among these genes (**Figure 7**), *TraesCS1A02G045300* and *TraesCS1A02G058400* were the most promising candidate genes associated with kernel weight, and their orthologs were *Os05g0115800* and *Os05g0121600* in rice, respectively. *Os05g0115800* was involved in the mitogen-activated protein kinase signaling pathway and was a mitogen-activated protein kinase phosphatase, affecting grain yield by regulating the grain number and grain size (Jiang et al., 2019). *Os05g0121600* was involved in the regulation of transcription, flower development, seed development, and endosperm development, and acted as a negative regulator in starch synthesis (Seetharam et al., 2021). In this study, we speculated candidate genes for TKW on chromosomes 4A and 5A. The physical interval of *TaTKW-4A* was 584.41–606.37 Mb, and 406 annotated genes were presumed. *TraesCS4A02G293900*, *TraesCS4A02G294000*, and *TraesCS4A02G303500* were the candidate genes for TKW, and the orthologs were *Os03g0669100*, *At4g34460*, and *At3g21510*, respectively. Among these genes, *Os03g0669100* and *At4g34460* encoded a regulator of G-protein signaling (RGB1 and AGB1) (Oliveira et al., 2022), and *At3g21510* (AHPs) and its encoded protein are related to the regulation of endosperm growth (Tran et al., 2021). RGB1, AGB1, and AHPs were involved in the regulation of grain traits (Li et al., 2019; Li et al., 2021). In *TaTKW-5A*, the physical interval was 436.63–481.93 Mb, and there were 512 high-confidence genes. *Os09g0448500* of the ortholog of *TraesCS5A02G233400* was a transcriptional regulator in rice and was associated with kernel traits (Li et al., 2019; Zhou and Xue, 2020). Consequently, *TraesCS4A02G293900*, *TraesCS4A02G294000*, *TraesCS4A02G303500*, and *TraesCS5A02G233400* were the candidate genes on 4A and 5A chromosomes in wheat (**Figure 7**). In *TaTKW-3B*, the physical interval was 7.29–31.66 Mb; a total of 418 annotated genes were found in the physical intervals, but we did not find orthologs related to TKW.

### 4.5 The major candidate gene expression of *TaTKW-1A*

Previous studies have shown that the growth of maternal tissues is able to control seed size through several signaling pathways, including the ubiquitin–proteasome pathway (Huang et al., 2017; Xie et al., 2018), G-protein signaling (Liu et al., 2018; Sun et al., 2018), mitogen-activated protein kinase signaling (Guo et al., 2018; Xu et al., 2018), phytohormones (Xu et al., 2015; Zhou et al., 2017), and transcriptional regulators (Wang et al., 2015; Segami et al., 2017). In our study, we found *TaTKW-1A*, which has two major candidate genes, *TraesCS1A02G045300* and *TraesCS1A02G058400*, with grain-related traits (**Figure 7**). We identified them in the transcriptome of wheat grain through the website https://www.ebi.ac.uk/gxa/home (Gillies et al., 2012; Li et al., 2013; Takafuji et al., 2021; Yu et al., 2021) (Figure S5). Results showed that both genes were expressed during grain development, although the expression profiles of these two genes clearly differ during grain development; both of them were expressed in the pericarp, endosperm, and seed coat (Li et al., 2013; Yu et al., 2021). Specifically, *TraesCS1A02G045300* is important because it was expressed consistently from anthesis to maturity (Pfeifer et al., 2014; Pearce et al., 2015; Yu et al., 2021).

### 4.6 KASP marker development

With the rapid development of marker-assisted selection in wheat breeding, the molecular marker technology has received increasing attention in recent years in crops (Song et al., 2022), and numerous markers have been developed (Kang et al., 2020; Rambla et al., 2022; Shin et al., 2022). A CAPS marker, *TaTPP-6AL1-CAPS*, was developed to differentiate *TaTPP-6AL1a* and *TaTPP-6AL1b*, which was associated with TKW (Zhang et al., 2017). A KASP marker of the candidate gene *TaFT-D1*, which was associated with TKW and KW, was developed and verified in a natural population (Liu et al., 2020b). Ten SSR markers for grain weight were developed and tested in 60 genotypes; all SSR primers had a high polymorphism (Sallam et al., 2019). In particular, numerous KASP markers for kernel traits have been developed in the last 2 years: *Kasp_5B_Tgw* for *QTgw.caas-5B* was developed and validated in wheat (Zhao et al., 2021), a KASP functional marker of *TaTAP46-5A* associated with kernel weight in wheat was developed and identified (Zhang et al., 2021), the KASP markers for *QTKW.caas-5DL* (Song et al., 2022), and the KASP markers for *QGl.cib-4A* (Li et al., 2022). These KASP markers provide a robust tool for genetic mapping and molecular breeding in crops. In this study, in order to use the advantage haplotypes, we developed the KASP markers of *TaTKW-1A*, which have been validated in the natural population. The results showed that the KASP markers could be used in wheat.

## 5 Conclusion

In this study, 48 QTLs were found in the RIL population, explaining 3.00%–33.85% of the phenotypic variances. Nine QTL clusters for kernel traits were identified in the RILs, and among these QTL clusters, we developed and validated the KASP markers of *TaTKW-1A*, and two candidate genes were predicted. The KASP markers and predicted candidate genes will be valuable for fine mapping and cloning the functional genes in wheat breeding.

## Data availability statement

The original contributions presented in the study are included in the article/Supplementary Material. Further inquiries can be directed to the corresponding author.

## Author contributions

CW (Chunping Wang) provided the test materials, performed the experiment, and revised the manuscript; ZZ participated in the trials, constructed the linkage maps, and wrote the paper. ZD participated in the development of the KASP markers. All authors read the final version of the manuscript and approved it for publication.
